# Harmonious ageing: a narrative review of music therapy in the biomedical literature

**DOI:** 10.1007/s41999-024-01146-z

**Published:** 2025-01-04

**Authors:** Shaz Raja, Ciara Barry, Rohit Upadhyay, Rana Alash, Méabh O’Raghallaigh, Róisín Hayes, Roman Romero-Ortuno

**Affiliations:** 1 School of Medicine, Trinity College Dublin, Dublin, Ireland; 2 Irish Association of Creative Arts Therapists, Dublin, Ireland; 3 Global Brain Health Institute, Trinity College Dublin, Dublin, Ireland; 4 Discipline of Medical Gerontology, Mercer’s Institute for Successful Ageing, St James’s Hospital, 6th Floor, Dublin 8, Ireland

**Keywords:** Healthy ageing, Music therapy, Biopsychosocial model, Physical health, Brain health, Social wellbeing

## Abstract

**Purpose:**

As the global population of older adults rises, the United Nations Decade of Healthy Ageing (2021–2030) advocates for disease prevention, management, and enhancing overall wellbeing in older adults. We reviewed the MEDLINE literature under the MeSH term “music therapy” (MT), for its role in promoting healthy ageing.

**Methods:**

A systematic search of the MEDLINE biomedical database (Ovid) was conducted using “MT” and “Ageing” as keywords, retrieving relevant full-text studies in English. Preference was given to more recent studies with higher levels of evidence. The studies were categorised according to the biopsychosocial framework into physical, cognitive and social domains, and further subcategorised based on their relevance to disease prevention and management.

**Results:**

The initial search identified 1147 articles, of which 75 met inclusion criteria. Studies encompassed both MT and music interventions delivered by non-music therapists. Overall, studies showed benefits in the promotion of physical health, including enhancing exercise capacity and improving fitness among older adults. Results were promising in managing conditions such as Parkinson’s disease and frailty. In the area of brain health, studies showed cognitive benefits, particularly in attention and processing speed among older adults. Socially, there was evidence of enhanced quality of life, reduced anxiety and depression, and improved social engagement, including in people living with dementia, underscoring the role of music in fostering emotional connections and mitigating caregiver stress.

**Conclusion:**

MT and interventions can enhance biopsychosocial health outcomes in older adults. Research should prioritise isolating MT’s specific effects, standardising definitions and methodologies, and exploring therapeutic mechanisms.

## Key summary points

**Aim:**

Review the MEDLINE literature under the MeSH term “music therapy” (MT), for its role in promoting healthy ageing and enhancing wellbeing across physical, cognitive, and social domains in older people.

**Findings:**

Our search retrieved studies that included both music therapy and music interventions delivered by non-music therapists.

**Message:**

Overall, the studies demonstrated benefits in physical fitness, cognition, and social functioning among older adults. Future research should prioritise isolating MT’s specific effects, standardising methodologies, and exploring therapeutic mechanisms to optimise its application.

## Introduction

The growing population of older people presents significant challenges for healthcare systems worldwide [1, 2]. In response to this demographic shift, the decade from 2021 to 2030 has been designated as the United Nations Decade of Healthy Ageing, with a primary objective of preventing and managing diseases while promoting overall wellbeing among older people [3]. One promising avenue for addressing the multifaceted needs of older individuals is through non-pharmacological interventions. The term “Music Therapy” (MT) was first introduced in 1974 as a Medical Subject Heading (MeSH) term in the US National Library of Medicine to signify “the use of music as an adjunctive therapy in the treatment of neurological, mental, or behavioural disorders”. In 2011, the World Federation of Music Therapy (WFMT) defined MT as “the professional use of music and its elements as an intervention in medical, educational, and everyday environments with individuals, groups, families, or communities who seek to optimise their quality of life and improve their physical, social, communicative, emotional, intellectual, and spiritual health and wellbeing” [4]. Currently, the British Association of Music Therapy (BAMT) defines MT as “an established psychological clinical intervention, delivered by registered music therapists to help people whose lives have been affected by injury, illness or disability through supporting their psychological, emotional, cognitive, physical, communicative, and social needs” [5].

While several reviews on MT exist, they often focus on specific aspects of its application, such as its use in dementia care [6] or physical rehabilitation [7]. Here, we reviewed the MEDLINE literature under the MeSH term “MT”, for its role in promoting healthy ageing, focussing on the most recent studies with the highest levels of rigour. Specifically, we integrated the included evidence within the biopsychosocial model to examine its effects on the physical, cognitive, and social functions of older people, both in prevention and management of age-related conditions. By applying the biopsychosocial model, this review sought to bring together insights that could help clinicians and others who serve older adults understand how MT can be applied in various settings. In addition, the review explored the extent to which MT, as a biomedical indexing term, reflects the pure definition of MT as outlined by professional bodies such as the BAMT.

## Methodology

### Search strategy

Articles were identified through the MEDLINE database via Ovid. The MeSH subject headings and keywords “Music Therapy” and “Ageing” were exploded, including all subheadings (none for MT), and combined using the “AND” operator to generate an initial list of articles. This search was conducted on February 16^th^, 2024, and included all articles registered up to that day (Table 1).

**Table 1:** Search strategy

Database: Ovid MEDLINE(R) ALL < 1946 to February 16, 2024 > Search strategy:	
**1** music therapy.mp. or exp Music Therapy/(5797) **2** exp Aging/ or Ageing.mp. or exp Aged/(3703278) **3** 1 and 2 (1147)	

Our aim was to review studies indexed under the specific MeSH term “Music Therapy” in MEDLINE, as this allowed us to focus on a well-defined and searchable term within the biomedical literature. MEDLINE is the US National Library of Medicine’s premier bibliographic database that contains more than 31 million references to journal articles in life sciences with a concentration on biomedicine. We chose “Music Therapy” because it is a recognised MeSH term (unique ID D009147), whereas “Music Intervention” is not available as a MeSH term, making it difficult to search systematically under that category. This means that not all the studies included in our review necessarily employed a formal, qualified music therapist as defined by professional bodies such as the BAMT. Rather, we were interested in understanding how the term “Music Therapy” has been used and applied within the biomedical literature, even if this usage does not always align with the strict definitions outlined by organisations like BAMT.

### Screening of studies

Articles were included if the study participants were older people (typically ≥ 65 years old), music was involved as therapy or intervention, and the outcome assessed at least one biopsychosocial parameter of health. Only full-text studies published in English were considered, and any lower-evidence studies (e.g. opinion pieces, non-research designs such as quality improvement projects, and small single descriptive studies) were excluded unless they provided significant insights within a specific theme.

### Selection of studies

Articles identified through the bibliographical search were retrieved from the database via the Library of Trinity College Dublin, Ireland. The five student reviewers divided the initial number of records into five equal groups, with each reviewer screening their allocated articles. Clearly irrelevant articles were excluded, and the remaining full-text articles were read for relevance. If any reviewer was uncertain about whether to include or exclude a study, a co-reviewer or the supervisor acted as a second reviewer to provide guidance. Any disagreements or uncertainties were discussed within the group, and the final list of articles included in the review was reached by consensus among all reviewers. Endnote® software was used to prevent duplication of research articles.

## Results

**Fig. 1:**
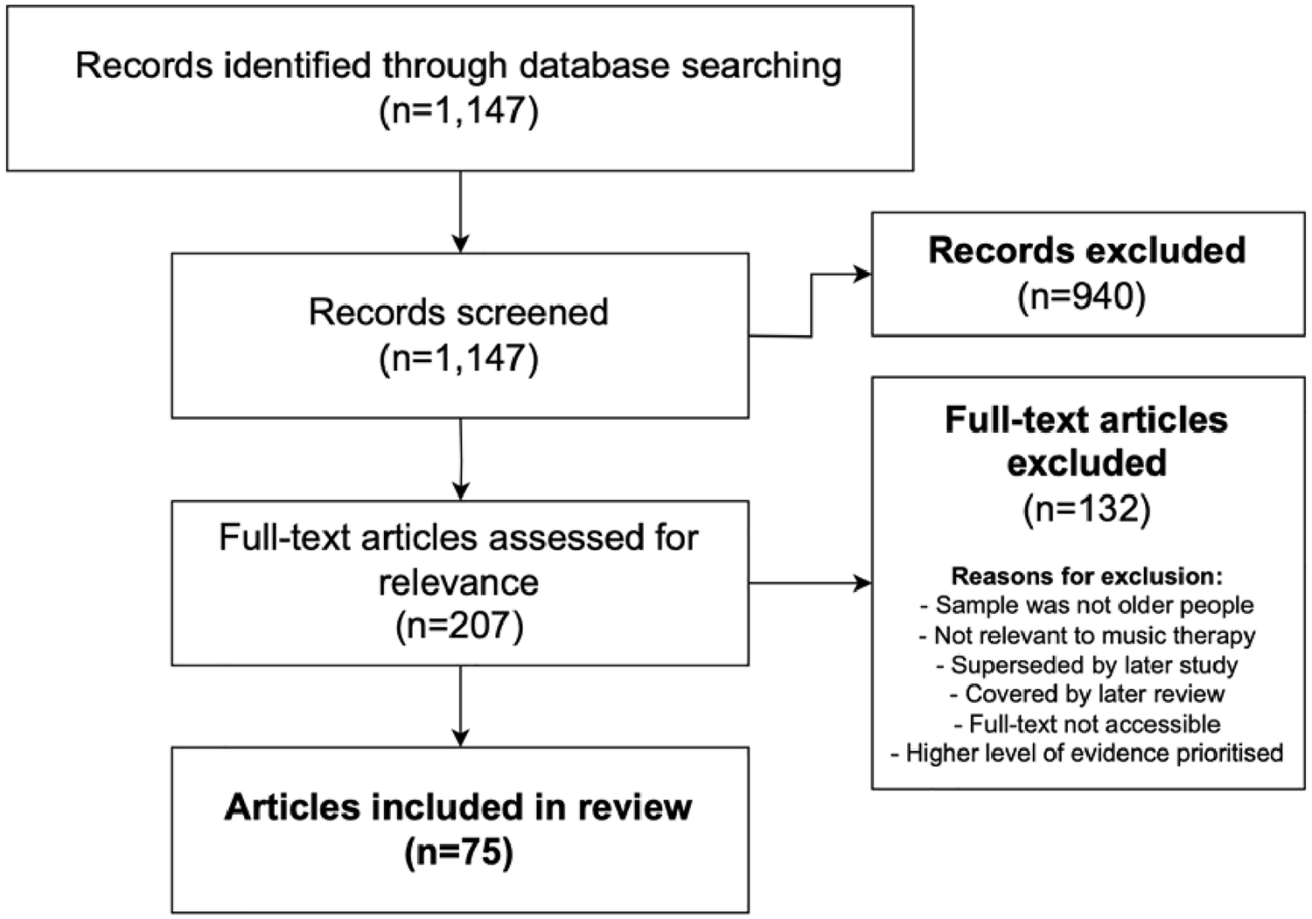
Flowchart of included studies

The initial search retrieved 1147 articles, of which 75 were finally included for thematic analysis based on a pre-agreed classification into physical, cognitive, and social domains, with further subdivisions into prevention and treatment within each category. The main reasons for exclusions are detailed in Fig. 1. The findings related to each domain and subcategories are narrated below.

### Physical health

#### Health promotion

The current guidelines from the World Health Organisation (WHO) recommend a weekly minimum of 150 min of moderate physical activity, highlighting its particular benefits for older adults [8]. This activity not only enhances physical health but also promotes social and mental wellbeing, thus promoting healthy ageing. A review of three randomised controlled trials assessed the effect of music listening on the physical activity of older adults [9]. Based on moderate-grade evidence, it was concluded that listening to music during a home-based walking exercise resulted in a small increase in the duration of physical activity, with a mean difference of an additional 52 min of moderate-level exercise per week. In addition, the study reported, with low-grade evidence, that music listening was associated with a 101-m increase in walking distance during a 6-min walk test. Overall, the review suggested that music listening serves as an accessible tool that effectively enhances the exercise capacity of older adults.

A Taiwanese study involving 146 community-dwelling older adults investigated the impact of a MT program on functional fitness [10]. *Kagayashiki*, a form of MT combining physical activity, was utilised to administer a fitness programme. The authors observed improvements in functional fitness in the intervention arm, which were assessed from baseline to 1 month and from baseline to 3 months (*n* = 133). Parameters observed to significantly improve included cardiopulmonary fitness, upper and lower body flexibility, upper limb muscle power, balance, and lower limb muscle endurance. A Japanese study assessing the effects of movement-based MT suggested that the intervention, even with a short duration (8 weeks), significantly improved balance, as well as systolic blood pressure and pulmonary function, in older individuals aged < 75 years old, compared to a control intervention without music [11].

With the aim of potentially enhancing postural reserve and reducing falls risk, Maclean et al. [12] investigated the effect of music training on gait patterns of healthy older adults. As walking becomes less automatic and requires more attention with increasing age, everyday activities such as talking while walking may pose a higher risk of falls in older people. Thus, the study examined gait in single-tasked (ST) and dual-tasked (DT) conditions. A DT “cost” was identified as gait slowing down when a cognitive task was simultaneously present. A total of 45 healthy older adults were divided into either a musical training group instructed to walk in time with the music, a music-playing group who simply walked while music was playing, or a no-music group who walked without music. Results concluded that musical gait training led to a significantly steadier gait in ST conditions and removal of the DT “cost”. These improvements were only observed in the musical training group, indicating that passive music listening while walking does not train gait.

#### Management of conditions

##### Frailty

A study by Sun et al. [13] conducted in 10 community care centres in southern Taiwan, analysed the therapeutic effects of MT with physical activity (MTPA) on conditions such as frailty (as identified by the Kihon Checklist), depression, physical fitness and cognitive function. With 122 older adults included, the intervention group (*n* = 62) received 12 weeks of MTPA with weekly 2-h sessions, while the control group (*n* = 60) participated only in socialising-based activities. The study found significant improvements in all tests following MTPA participation, while the control group’s performance in the fitness test diminished, potentially due to lower physical activity. The authors concluded that MTPA may be effective in improving frailty and depression, as well as physical fitness and cognitive function more generally. Another study by Murabayashi et al. [14] utilising a randomised controlled crossover trial design found that in 94 older people who had been identified as frail by the Kihon Checklist, MT sessions conducted for 45–50 min once a week for 12 weeks significantly improved performance in physical function (Timed Up and Go: TUG) and psychological health (Geriatric Depression Scale-15, General Health Questionnaire-12).

##### Motor symptoms of Parkinson’s disease (PD)

In research on PD, and without a formal focus on MT, Brown et al. [15] observed in 2009 that, in a study involving 10 older PD cases and 10 controls, gait among the PD participants was adversely affected by concurrently listening to self-selected music. However, an MT-focussed meta-analysis in 2021, evaluating 17 studies with 598 participants, aimed to determine the effect of music-based movement therapy on clinical outcomes in people living with PD [16]. The analysis revealed that motor function significantly improved in nine studies using the TUG test. Balance also improved in eight studies using the Berg Balance Scale and three studies using the Mini-Balance Evaluation Test. In addition, walking speed significantly increased in seven of the included studies. The authors concluded that MT could have a positive impact on the physical function of older adults living with PD.

##### Cardiovascular disease

A study evaluated the effect of MT on the quality of life (QoL) and blood pressure (BP) control in hypertensive patients over 50 years old. Participants were divided into an experimental group (*n* = 23), which received weekly MT sessions for 12 weeks in addition to conventional treatment, and a control group (*n* = 24), which received only standard treatment. The MT group showed significant improvements in both QOL and BP control compared to the controls, suggesting that MT can be a beneficial adjuvant in the multidisciplinary care of hypertensive patients [17]. In a study by Okada et al. conducted in 2008 involving 87 hospitalised older patients with pre-existing cerebrovascular disease and dementia [18], the intervention group receiving MT (*n* = 55) showed a reduced susceptibility to developing congestive heart failure. This outcome was attributed to evidence of improved heart rate variability (HRV) and reduced levels of plasma catecholamines (adrenaline, noradrenaline), and interleukin 6. The authors discussed these findings in the context of improved parasympathetic autonomic tone in the MT group.

##### Pain

A randomised clinical trial investigated the impact of music on osteoarthritis pain in older adults. Participants who listened to music for 20 min daily experienced significantly less pain compared to those who sat quietly [19]. A meta-analysis of 97 RCTs from 1995 to 2014 examined the effect of music on pain, with results showing significantly reduced pain intensity, emotional distress, anaesthetic and analgesic use, heart rate, and blood pressure [20]; however, a limitation was the inclusion of a mix of music interventions and what could be formally classified as MT. Likewise, a systematic review by Hsu et al. published in 2022 examined studies on music interventions for chronic pain in older adults. Eight studies involving 524 participants found positive outcomes, though small and short-term, in reducing chronic pain [21].

A study by Wang et al. evaluated perioperative psychological and music interventions in older surgical patients. Forty patients were randomised into an intervention and a control group. The intervention group showed significant reductions in anxiety and postoperative pain, with improved HRV [22]. Furthermore, a systematic review assessed the effect of music on postoperative recovery in cancer patients aged 60 and older, and results showed that music reduced pain and anxiety, and improved relaxation, cognitive function, and patient satisfaction, with no negative side effects [23].

A study investigated music’s effect on pain in home-dwelling persons living with dementia. Participants listened to preferred music before their predicted ‘peak agitation time’, twice weekly, and pain levels (measured with the M-PADE tool [24]) significantly decreased after listening to music [25]. A multicentre randomised controlled trial compared the efficacy of choral singing versus painting sessions on chronic pain, mood, quality of life, and cognition in mild Alzheimer’s disease (AD) patients. Both interventions significantly reduced pain, anxiety, and improved quality of life and cognitive abilities [26].

##### Sleep

A 2021 systematic review assessed the efficacy of music interventions on sleep quality in older adults [27]. Nine studies were included, with only one examining active music intervention and the rest utilising passive music listening as the intervention. Meta-analysis of six studies using the Pittsburgh Sleep Quality Index indicated that music intervention might have a positive effect on sleep latency, duration, and efficiency.

A 2023 randomised clinical trial examined the effects of tailored music intervention on the sleep quality of older adults with a dementia diagnosis or self-reported memory impairments [28]. The music genre was tailored to the likes of the participant and the 30-min playlist was played at bedtime for 1 month. Although the subjective experience of the participants was positive, only a third of their caregivers felt that the experiment improved the participant’s sleeping habits. In addition, the quantitative measures of sleep latency, wake-after-sleep-onset and total sleep duration yielded statistically non-significant results.

### Brain health

#### Health promotion

Playing a musical instrument requires the brain to receive and process auditory, visual, motor, and somatosensory information and involves the interaction of higher order cognitive functions. Reflecting on these mechanisms, Wan and Schlaug suggested in 2010 that music training could be a beneficial intervention for preserving brain health in ageing [29].

In a secondary analysis of a randomised controlled trial published in 2014, Hars et al. investigated the effects of 6 months of music-based multitask training on cognitive functioning and mood in older adults. Participants, aged ≥ 65 years and at increased risk for falling, were randomly assigned to either an intervention group attending weekly supervised multitask exercises to piano music rhythm or a control group maintaining usual lifestyle habits. Results showed improvements in sensitivity to interference (one aspect of executive function) and reduced anxiety levels in the intervention group compared to controls. In addition, within-group analysis revealed increased Mini-Mental State Examination (MMSE) scores and fewer participants with impaired cognitive performance in the intervention group [30].

A 2015 systematic review and meta-analysis assessed whether MT affects cognitive function in older adults. Five studies with 234 participants (mean age per study of 71–82 years) were included. The analysis focussed on short-term cognitive outcomes, specifically using MMSE data. While some individual studies suggested significant cognitive improvements from active MT, the meta-analysis revealed no significant short-term effects overall. The study recommended future research with better methodologies and long-term designs [31]. A 2017 review by Klimova et al. suggested that music interventions, alongside physical activities such as walking and aerobic exercises, adherence to the Mediterranean diet, and engaging in cognitive activities like solving crosswords, showed promise as potential interventions against cognitive decline in normal ageing [32].

In 2024, a systematic review and meta-analysis examined the impact of music and rhythm interventions on cognition in adults aged 60 and older. Analysing thirteen studies with 502 participants, the findings indicated that musical instrument training improved attention inhibition, switching, and processing speed. The authors argued that since these aspects of cognition exhibit age-related decline, beginning learning to play a musical instrument in adulthood may contribute to cognitive preservation in later life [33].

#### Management of conditions

##### Cognitive impairment

In a randomised trial, Biasutti and Mangiacotti studied the impact of a cognitive training program using rhythm-music and improvisation on older adults’ executive functions. Thirty-five participants including cases of mild–moderate cognitive impairment were randomly assigned to either a group receiving cognitive music training or a control group doing gymnastics. The experimental group showed significant improvements in cognitive function measures, such as the MMSE, verbal fluency test, and clock-drawing test, while the control group did not [34].

Another study explored the effects of art therapy (AT) and music reminiscence activity (MRA) on cognitive functions in older people with mild cognitive impairment (MCI), compared to a control group. Results showed that AT improved neurocognitive domains at 3 and 9 months, but changes in depression, anxiety, and telomere length were non-significant [35].

In 2022, Jordan et al. reviewed nine studies (8 RCTs, 1 observational) exploring music interventions for individuals with MCI suggesting that such interventions may be beneficial in improving symptoms and potentially delaying progression to dementia [36]. In a further 2023 study, Kim et al. systematically reviewed the impact of music interventions on cognitive function in older adults with MCI. A search across multiple databases yielded 11 relevant articles. The analysis revealed that music interventions significantly enhanced global cognitive function, verbal fluency, executive function, and spatial function in this population. However, the studies varied in intervention type, cognitive assessment tools, and duration, with some showing bias risks due to missing data and confounding factors [37].

##### Dementia

Many studies have investigated the possibility that music interventions may help in the management of behavioural and psychological symptoms of dementia (BPSD) [38–44]. In a 2013 systematic review and meta-analysis, Ueda et al*.* revealed moderate effects on anxiety and small effects on behavioural symptoms [45]. A 2014 meta-analysis [46] was in keeping with these findings but noted inconclusive long-term outcomes, suggesting that music interventions may only be beneficial short-term for BPSD. Two further 2015 reviews found that while group music interventions showed promise in reducing dementia-associated anxiety, the limited number of studies, varied methodologies, and definitions hindered definitive conclusions [47, 48]. In a 2017 systematic review and meta-analysis, Zhang et al. investigated MT’s potential as a primary non-pharmacological treatment for older patients living with dementia. Through a comprehensive analysis of 34 studies involving 1757 subjects, they found significant improvements in BPSD, particularly in studies including interactive MT sessions, defined as recipients being actively engaged in making music and singing [49]. In 2018, a systematic review of reviews examined non-pharmacological approaches to managing BPSD in older adults, finding that individual, sensory-focussed interventions, such as MT and pain-targeted approaches, showed the most conclusive benefits [50].

A 2017 Cochrane review on music‐based therapeutic interventions for people living with dementia analysed 17 studies involving 620 participants with varying degrees of dementia residing in institutions. While most interventions combined active and receptive musical elements, the methodological quality of the studies varied, with all at high risk of performance bias and some at high risk of detection or other biases. The findings suggested low-quality evidence that music-based therapeutic interventions may have little to no effect on emotional wellbeing, quality of life, overall behaviour problems, and cognition, with moderate-quality evidence indicating a reduction in depressive symptoms but no decrease in agitation or aggression. However, the evidence for long-term outcomes, anxiety, and social behaviour was of very low quality, with the authors calling for further research [51].

A study examined the effects of a 6-week music-with-movement (MM) intervention on the cognitive functions of people with moderate dementia, comparing it to music listening (ML) and social activity (SA) interventions. Delivered by non-music therapists using recorded music, the MM intervention led to greater improvements in memory and depressive symptoms compared to the other groups. However, mixed multivariate analysis showed no significant group-by-time interactions, indicating insufficient evidence for long-term differences between the interventions [52]. In a controlled randomised single-blind study, cognitive training significantly improved initiative (a basic executive function interposed between motivation and the planning of goal-directed actions) in mild-to-moderate AD patients; however, active MT delivered by a music therapist and neuroeducation were not associated with changes in initiative [53].

However, in a 2023 study, Bleibel et al. systematically reviewed eight randomised controlled trials, concluding that MT, particularly active music intervention, improved cognitive functions in patients with AD compared to control groups [54].

##### Anxiety

In 1998, Chlan conducted a study testing a ‘nonpharmacologic nursing intervention’ on relaxation and anxiety in alert, non-sedated, ventilated intensive care unit patients. Fifty-four patients were randomised to either listen to a chosen tape through headphones for 30 min or have a rest period. The music group reported significantly less anxiety and exhibited decreased heart and respiratory rates compared to the control group [55].

A 2017 review by Istvandity focussed on intervention studies concentrating on music reminiscence therapy, which incorporates both music and reminiscence activities. Analysing studies published between 1996 and 2016, the review included five studies predominantly conducted in aged care facilities. Positive effects were observed in four out of five studies, predominantly the reduction of anxiety, depression and stress [56].

A 2022 systematic review suggested the effectiveness of music as an intervention for the reduction of anxiety during normal care delivery in adult hospitalised patients [57].

##### Depression

Two studies explored the impact of music on depressive symptoms in older individuals. The first study examined the effects of interactive group MT versus recreational group singing on older nursing home residents, finding that MT significantly reduced depressive symptoms more effectively than recreational singing over a 12-week period [58]. The second study compared the effects of stimulative and sedative music videos (MVs) on depressive symptoms and physiological relaxation in older adults with depressive symptoms. It found that both types of MVs improved depressive symptoms and physiological relaxation, with stimulative MVs being particularly effective [59].

Two randomised controlled trials investigated the impact of music on depression and physiological parameters in older individuals. The first study in Hong Kong found significant decreases in depression, blood pressure, heart rate, and respiratory rate among 23 older participants after 1 month of listening to recorded music for 30 min per week [60]. The second study, conducted in a Turkish nursing home, revealed that music therapist-delivered MT over 8 weeks significantly reduced depression levels and systolic blood pressure in 64 older residents [61]. One study by Yu et al. in 2022 demonstrated depression-reducing effects of a nurse-delivered group music intervention on older people in two nursing homes. Results revealed a statistically significant difference in depression (Chinese version of the Geriatric Depression Scale) between weeks 5 and 10 in this randomised control trial [62].

A systematic review and meta-analysis of 19 randomised controlled trials found that MT, combined with standard treatment, significantly reduces depressive symptoms in older people (standardised mean difference = 1.02; 95% CI 0.87, 1.17). Despite these promising results, the review concluded that high-quality trials are still needed to further evaluate the effects of MT on depression [63].

### Social health

#### Health promotion

##### Quality of life

In 2004, case reports by Suzuki et al. revealed that patient A (an 86-year-old man with vascular dementia) did not take initiative or speak voluntarily before an MT intervention. However, he had an emotional reaction to the first music session and voluntarily participated in every music activity and positively interacted with the nurses. Patient B (right-sided paralysis) did not speak voluntarily before MT started; however, by the fifth session, she smiled and greeted the nurses. As sessions progressed, her expressiveness increased, she actively participated and during the sessions she talked with the other participants [64].

It has been argued that music interventions represent a non-invasive, straightforward, and cost-effective therapeutic approach to enhancing the quality of life for older individuals living in the community [65]. In 2010, a study by Travers and Bartlett evaluated the impact of Silver Memories, a radio programme targeting social isolation and loneliness in older individuals by broadcasting musical content from the 1920s to 1950s. Results indicated significant improvements in quality of life and depressive symptoms among participants over 3 months; however, social isolation and loneliness did not improve. The initial low level of social isolation may have left little room for improvement [66]. Solé et al. noted that various forms of music interventions (choir, music appreciation and preventive MT sessions) had positive effects on the self-reported quality of life of a sample of 83 healthy older adults [67]. They revealed that the main motivation for participation of older adults in musical activities were social reasons—to ‘be amongst pleasant people,’ and ‘to make friends’, as well as cognitive reasons. Open questionnaires revealed that the most enjoyable component of musical activities was the music itself, and the comradeship generated. Although results revealed no significant changes in quality of life, the participants’ subjective perception was that involvement in these musical interventions improved some components of their quality of life, especially in personal development and in social relations (e.g. making more friends).

#### Management of conditions

##### Social isolation and loneliness

Older people living alone or in nursing homes can be faced with social isolation and loneliness, and music interventions can be utilised to combat them [68, 69]. A study by Biasutti and Mangiacotti, involving a music training programme designed to analyse its effectiveness on depressed mood on older people with or without cognitive decline revealed a significant improvement in depressive symptoms (Geriatric Depression Scale) for the experimental period compared with control group. Furthermore, the music training also appeared to have positive effects in relation to social interaction among group members. Fifty-five percent of the participants in the experimental group considered the training an opportunity to ‘interact’ and ‘make new friendships’ in semi-structured interviews. As well as this, 64% of the participants reported that the music training helped them to be more ‘active’ in their daily life activities. Overall, analysis of these interviews showed that the music training helped participants to establish new relationships while improving mood [70].

Dassa and Amir demonstrated that carefully selecting music from the participants’ past can encourage conversation among people with middle to late-stage AD, despite language deficits. Due to the language deterioration associated with AD, conversation can become burdensome for patients. However, the group singing of songs from the participants past stimulated spontaneous discussion and allowed participants to connect with each other [71].

A randomised clinical crossover trial by Reschke-Hernandez et al. involving 32 care facility residents with AD and other dementias revealed significant positive effects of MT on social engagement, especially for those with moderate dementia. On average, participants demonstrated more constructive engagement during MT compared to during verbal sessions (the analogous non-music condition, control group). Participants demonstrated more laughter, smiling and affectionate behaviour during MT [72].

A pilot study by Shah-Zamora et al. investigated the effects of virtual group MT for apathy in PD. The reduced interest caused by apathy can lead to social withdrawal in PD patients. However, the post-intervention satisfaction survey revealed that the top three most enjoyed features of the MT were interaction with the music therapist, social interaction, and musical activities. Results also revealed marked improvement in apathy [73].

##### Caregiver stress

In their 2018 review, Elliott and Gardner concluded that enhanced social wellbeing is one of the ways in which music can positively influence the lives of older adults living with dementia in the community. Specifically, music was found to support social relationships, strengthen the caregiver relationship, and increase opportunities for interaction, connection, and engagement with others [74].

A study by Gotell et al. illustrated the reactions and social interactions of older people with dementia or suspected dementia and their caregivers before, during and after music events. Results revealed that immediately after the music events, some of the patients were quite talkative, attempting to converse with one another. Caregivers also experienced bonding with their care receivers [75].

Clair et al. examined the effects of caregiver-implemented music applications on engagement with their care receivers. Eight caregiver-care receiver pairs participated in sessions where a music therapist trained caregivers to use a music application. The study found a highly significant increase in engagement frequency over five sessions, concluding that MT applications effectively enhance mutual engagement in caregiving couples affected by dementia [76]. In a randomised control trial by Sarkamo et al., the caregivers in both music intervention groups (listening coaching group and singing coaching group) rated the musical activities as being both highly beneficial to themselves as well as for their interaction with the patient with dementia. Music listening also had a positive effect on the quality of life of the patients living with dementia [77].

Another study found that staff perceptions of personalised music for people with dementia in residential aged care were generally positive, with themes of reminiscence and optimism emerging. However, staff also cited logistical challenges, such as limited availability [78].

## Discussion

This narrative literature review including 75 studies (plus nine additional background references) examined research on the use of music as a tool to promote healthy ageing and enhance wellbeing in older populations. Findings were categorised into physical, cognitive, and social health domains within a biopsychosocial framework. Focussing our search on the term ‘MT’ in MEDLINE revealed that the included studies encompassed both MT and music interventions delivered by non-music therapists. As such, this review offers a primer and practical guide for clinicians new to the field.

### Main findings

Music interventions support physical health in older adults by enhancing fitness, cardiovascular health, and managing conditions such as frailty, motor symptoms of PD, and pain. Furthermore, music can reduce pain intensity and emotional distress, and shows potential to improve sleep quality, though further research is needed to confirm these effects.

Music interventions can positively impact brain health, cognitive function, and emotional wellbeing in older adults, especially those at risk of cognitive decline or living with dementia. Evidence suggests that playing instruments and music-based multitask training can enhance cognitive functions and reduce symptoms of anxiety and depression. For individuals living with dementia, music interventions, including rhythm and reminiscence activities, can improve behavioural and psychological symptoms, though more research is needed on long-term effects. In addition, MT shows promise in reducing depressive symptoms and anxiety, supporting its role as a complementary, non-pharmacological therapeutic approach for older adults.

Music interventions can enhance social health by improving quality of life, reducing social isolation, and strengthening caregiver relationships among older adults, particularly those living with dementia. Studies show that MT can encourage social engagement, foster new friendships, and deepen bonds with caregivers. Specific interventions, such as virtual group MT and personalised music, have been effective in reducing apathy in PD and encouraging positive social behaviours in dementia care settings. Despite logistical challenges and reduced availability in many settings, MT is well evidenced as a valuable approach for enhancing social wellbeing and caregiver satisfaction in the care of older adults.

### Potential mechanisms

The literature explores several potential physiological mechanisms underlying the therapeutic effects of music, particularly in older adults living with dementia. Four key theories propose different pathways through which music may exert its effects: (1) as a psychological distraction reducing stress and enhancing coping mechanisms; (2) as a physiological distraction diverting sensory input; (3) as a stimulant releasing endorphins and modulating catecholamine levels for pain relief and cardiopulmonary functions; and (4) as a modulator decreasing sympathetic tone to alleviate stress responses. Empirical evidence supports the cognitive-behavioural framework model where preferred music schemas mediate behavioural effects, particularly in reducing anxiety and promoting relaxation. In addition, certain types of music may be more effective than others [79]. However, studies on biomarkers such as cortisol levels, heart rate, and blood pressure show mixed results, necessitating further research [80]. Future investigations should also explore how music influences psychobiological pathways to fully understand music’s therapeutic potential [81].

### Methodological limitations

Our review faced several limitations. Restricting the search to a single biomedical database likely led to omissions, particularly of studies in non-biomedical databases or grey literature. We included only studies at the cohort level or higher, with lower-level studies reviewed cautiously in areas with limited evidence, such as social health. Priority was given to studies post-2000 for recent evidence, though relevant older studies were referenced. The search was limited to English-language articles, possibly excluding important research in other languages. Finally, as noted above the “MT” term in MEDLINE encompassed both MT and non-MT studies.

Multi-component interventions make it challenging to attribute benefits solely to music, as effects may stem from other elements [82, 83]. In dementia research, frameworks like the progressively lowered stress threshold model and Kitwood’s theory of personhood are often overlooked [84]. Protocol variability complicates study comparisons, particularly in meta-analyses. Subjective measures (pain, sleep, and mood) depend on self-report, adding variability, while cognitive outcomes are hard to standardise due to diverse participant criteria and tools. Individual musical preferences may influence outcomes through reminiscence, and the social interaction in music sessions could independently contribute to positive effects.

### Music therapy vs. music interventions

Our review highlighted that the MeSH term “MT” in MEDLINE retrieved a diverse range of music intervention studies, many of which were not conducted by professional music therapists. While this broad categorisation reflects the variety of music interventions in older adults’ healthcare, it may lead to variations and potential confusion in reported outcomes. Interventions without trained music therapists could benefit from professional expertise; for example, music therapists use rhythmic auditory stimulation to align music tempo with gait, with live music allowing for dynamic adjustments that enhance safety and effectiveness. We recommend that biomedical databases and future research distinguish between formal MT and other music interventions to help clarify the specific therapeutic benefits of professionally guided MT.

## Conclusion

This review of the main biomedical database underscores the valuable role of MT and various music interventions in promoting healthy ageing across physical, cognitive, and social health domains. By highlighting evidence-based benefits, this review serves as a practical guide for clinicians, especially those new to the field. Although music interventions offer a promising, non-invasive approach to enhancing wellbeing in older adults, further research is essential to distinguish between the effects of professionally guided MT and other music interventions. Standardising methodologies and exploring underlying therapeutic mechanisms will be key to maximising MT’s impact and ensuring it becomes a widely accessible resource for supporting holistic health in older populations.

## Data Availability

This study is a literature review and does not involve the generation or analysis of new datasets. All data referenced in this review are available through the cited studies and sources.
